# Spatial and temporal disease dynamics of the parasite *Hematodinium* sp. in shore crabs, *Carcinus maenas*

**DOI:** 10.1186/s13071-019-3727-x

**Published:** 2019-10-11

**Authors:** Charlotte E. Davies, Frederico M. Batista, Sophie H. Malkin, Jessica E. Thomas, Charlotte C. Bryan, Peter Crocombe, Christopher J. Coates, Andrew F. Rowley

**Affiliations:** 1 Department of Biosciences, College of Science, Swansea University, Swansea, SA2 8PP Wales UK; 2 Present Address: Centre for Environment Fisheries and Aquaculture Science (CEFAS), Weymouth, Dorset UK

**Keywords:** *Hematodinium*, Endoparasites, *Carcinus maenas*, Disease connectivity, eDNA, Aquatic vectors, Fisheries, Invasive species

## Abstract

**Background:**

The parasitic dinoflagellates of the genus *Hematodinium* represent the causative agent of so-called bitter or pink crab disease in a broad range of shellfish taxa. Outbreaks of *Hematodinium-*associated disease can devastate local fishing and aquaculture efforts. The goal of our study was to examine the potential role of the common shore (green) crab *Carcinus maenas* as a reservoir for *Hematodinium*. *Carcinus maenas* is native to all shores of the UK and Ireland and the North East Atlantic but has been introduced to, and subsequently invaded waters of, the USA, South Africa and Australia. This species is notable for its capacity to harbour a range of micro- and macro-parasites, and therefore may act as a vector for disease transfer.

**Methods:**

Over a 12-month period, we interrogated 1191 crabs across two distinct locations (intertidal pier, semi-closed dock) in Swansea Bay (Wales, UK) for the presence and severity of *Hematodinium* in the haemolymph, gills, hepatopancreas and surrounding waters (eDNA) using PCR-based methods, haemolymph preparations and histopathology.

**Results:**

Overall, 13.6% were *Hematodinium*-positive *via* PCR and confirmed *via* tissue examination. Only a small difference was observed between locations with 14.4% and 12.8% infected crabs in the Dock and Pier, respectively. Binomial logistic regression models revealed seasonality (*P* < 0.002) and sex (*P* < 0.001) to be significant factors in *Hematodinium* detection with peak infection recorded in spring (March to May). Male crabs overall were more likely to be infected. Phylogenetic analyses of the partial ITS and *18S* rRNA gene regions of *Hematodinium* amplified from crabs determined the causative agent to be the host generalist *Hematodinium* sp., which blights several valuable crustaceans in the UK alone, including edible crabs (*Cancer pagurus*) and langoustines (*Nephrops norvegicus*).

**Conclusions:**

Shore crabs were infected with the host generalist parasite *Hematodinium* sp. in each location tested, thereby enabling the parasite to persist in an environment shared with commercially important shellfish.
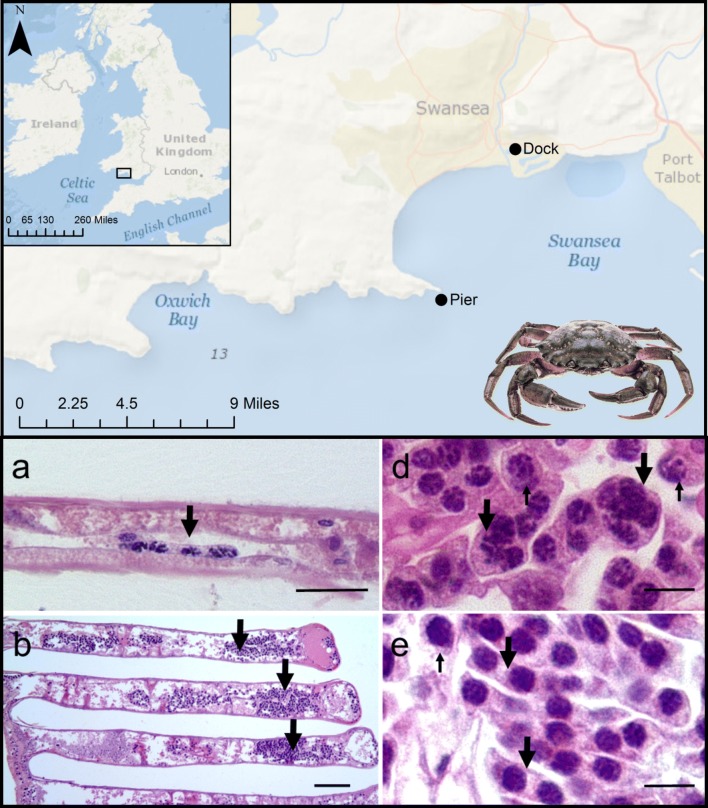

## Background

The dinoflagellate endoparasites of the genus *Hematodinium* are an important group of disease-causing agents infecting over 40 species of crustaceans worldwide [1]. They were first discovered in the 1930s infecting shore crabs (*Carcinus maenas*) in northern France but only at low prevalence and the causative agent was named *Hematodinium perezi* [2]. Species of *Hematodinium* have been recorded in several commercially important species of crustaceans and considered a major cause of loss of stocks. In Virginia (USA), loss to the blue crab fishery due to infection can exceed USD 500,000 per year in non-epidemic years [1]. Similarly, losses to the Norwegian lobster (*Nephrops norvegicus*) fishery based on the west coast of Scotland (UK) have been estimated to be of the order GBP 2–4 million [3–5]. In another key economic crustacean, the edible crab (*Cancer pagurus*), infection levels of up to 30% have been reported in juveniles (pre-recruit) in the Bristol Channel (UK), indicating that this infection alone can cause significant reduction in stocks [6]. In France, the fishery for the velvet swimming crab (*Necora puber*) suffered huge losses from 1984–88 (> 96 %) due to *Hematodinium* infection [7]. In North America, outbreaks have infected up to a third of the Tanner crab (*Chionoecetes bairdi*) and snow crab (*C. opilio*) stocks in southeast Alaska and Newfoundland, respectively [8, 9].

*Hematodinium* spp. have been reported to have seasonal, sex and size related relationships or correlations with their hosts that vary depending on the host species and location [4, 10, 11]. For example, in studies of *C. maenas*, the prevalence of *Hematodinium* infection peaked in April, and was significantly higher in males than in females [10]. However, in *N. norvegicus*, peak infection prevalence occurred during the winter [12] and was highest in smaller individuals and females [4]. The timescale of infection by *Hematodinium* spp. from initial contact through to host death is also highly variable and probably related to host, geographical location and the parasite’s genotype [13]. In pre-recruit edible crabs (*C. pagurus*), infection likely occurs in the latter part of the year between October and December [6]. It can take up to one year for the host to die either due to multiplication of *Hematodinium* in the haemolymph resulting in metabolic exhaustion [1, 14] or due to co-infections [15]. Environmental DNA (eDNA) is increasingly being used to detect the molecular ‘signatures’ of pathogens in the absence of, or before entry into, a host (i.e. the water column) [16]. Detection of *Hematodinium* spp. in eDNA samples prior to host contact has led to a previously un-reported stage in the parasite life-cycle being suggested [17, 18].

The common shore crab (or green crab), *C. maenas*, is found on all coasts of the UK and Ireland, predominantly in the neritic zone but also at depths greater than 60 m. Although native to the North-East Atlantic from northern Norway southwards to West Africa, it has been introduced to the USA, Sri Lanka, the Red Sea, Madagascar, South Africa and Australia. It is considered to have damaging effects on indigenous species [19, 20]. Shore crabs tolerate a wide range of salinities and temperatures and their establishment in such a diverse range of environments, shared with other important commercial species, makes it an essential subject for disease research. This species is known to harbour a wide range of parasites and pathogens, including *Hematodinium* spp. [21]. Fisheries for *C. maenas* occur in Spain, France and Portugal, where hundreds of tons per year of intermoult crabs are exported [22, 23]. Additionally, over one million crabs are removed annually from estuaries in the UK to be sold as bait [24]. In the USA, ovigerous crabs are used as bait for both conch and fish species [25]. The invasive and adaptive nature of this species alongside its extensive use as bait presents a clear rationale for the monitoring of pathogens, which in turn may aid in the management of species of commercial importance [26, 27] and help to predict ecosystem functioning [28].

Here, we investigated the presence of *Hematodinium* spp. in *C. maenas* across two contrasting locations in South Wales, UK. These locations represent habitats shared with commercially important species of crabs including the edible crab (*C. pagurus*) and velvet swimming crabs (*N. puber*). We monitored the presence of this parasite in crab tissues using histology (e.g. gill, hepatopancreas) and PCR (haemolymph). Additionally, we isolated eDNA from the surrounding waters in order to assess fully the spatial and temporal prevalence of patterns of *Hematodinium* spp.

## Methods

### Study area

**Fig. 1:**
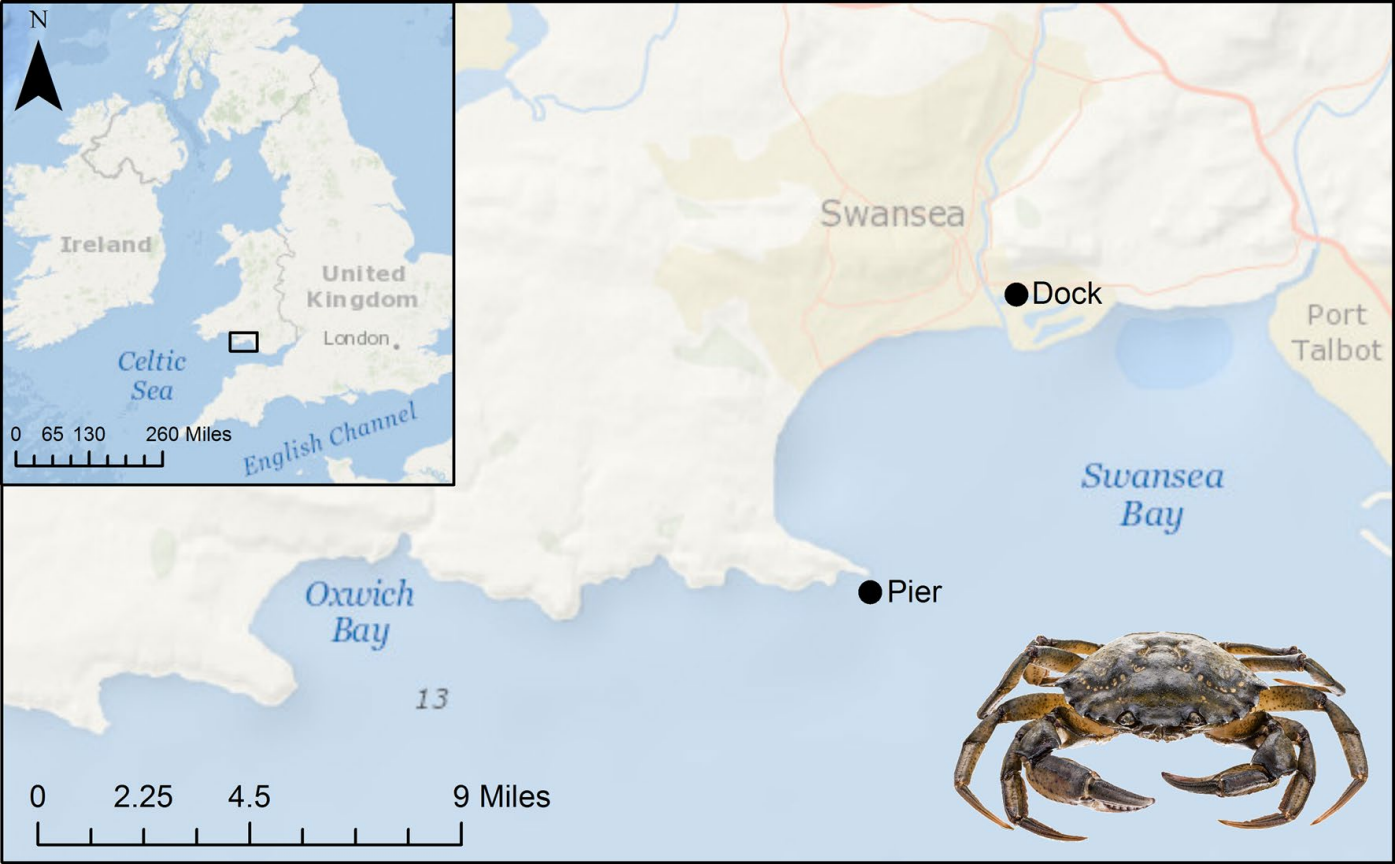
Collection locations for shore crabs (*Carcinus maenas*) used during this study, South Wales, UK. Maps were created and annotated in ArcGIS v.10.5.1 (Service Layer Credits: Sources: Esri, GEBCO, NOAA, National Geographic, Garmin, HERE, Geonames.org, and other contributors)

The study took place off the South Wales coast, UK at two distinct locations. The first location, the Prince of Wales Dock, Swansea (51°37′8.76″N, 3°55′36.84″W), is a mostly disused 27-acre dock to the east of the River Tawe (Fig. 1). The second location, Mumbles Pier (51°34′8.958″N, 3°58′33.297″W), is an intertidal rocky shore to the south of Swansea Bay (Fig. 1) facing into the Bristol Channel with a twice daily tide ~ 8.5 m in height.

### Sample collection

Once per month, for 12 months from November 2017 to October 2018, the shore crab population was surveyed at both locations. Strings of baited Swedish crayfish traps were deployed and immersed for 24 h, retrieved and 50 crabs were chosen randomly, bagged individually and transported back to the laboratory on ice. In addition, for environmental DNA analysis, three 2-l bottles of seawater from each location were sampled and transported on ice back to the laboratory. In the Dock location, pots were deployed from the pontoon, and water was taken directly from 3 replicate sites (*c*.60 m apart) across the dock pontoon. In the case of the Pier location, pots were deployed and collected from around the base of the Pier at low tide, and a research vessel was used to collect water from 3 replicate sites adjacent to the Pier (*c*.60 m apart). Water samples were collected from each location for the same 12-month period as the crab sampling, with the exception of December 2017 from the Dock location, which is absent from the data set.

### Laboratory regime

All crabs were processed on the day of collection. The following biometric data for each crab were taken: carapace width (CW; mm); sex; moult stage [inter-moult (hard) or post-moult (soft)]; fouling (presence of epibionts); pigment loss or shell disease; limb loss; and carapace colour (green, yellow or orange/red). In addition, *c.*350 μl of haemolymph was withdrawn using a 23-gauge hypodermic needle fitted with a sterile 1-ml syringe and haemolymph appearance was categorised as clear or milky. Haemolymph was fixed 1:1 with 25 µl of 5% formaldehyde (v/v) in 3% NaCl (w/v) solution and total haemocyte counts were recorded using an improved Neubauer haemocytometer under phase contrast microscopy. A further 25 µl of haemolymph was placed onto a microscope slide for primary screening using the phase contrast optics of a BX41 microscope (Olympus, Tokyo, Japan), and 100 µl was stored at − 80 °C for subsequent DNA extraction. If a haemolymph preparation was deemed *Hematodinium-*positive, the number of parasites/ml haemolymph was calculated as a marker of severity by counting the total number of haemocytes in a haemocytometer and determining the ratio of *Hematodinium* to haemocytes in haemolymph preparations.

### Water filtration

All water samples were processed in the laboratory on the same day as collection. Two litres of water from 3 replicate sites at each location was first filtered through a sterile 200 μm nylon mesh to remove large debris. Next, the water was vacuum filtered through a sterile 0.45 μm (pore size), 47 mm (diameter) polyvinylidene difluoride (PVDF) Durapore^®^ membrane filter (Sigma-Aldrich, Dorset, UK) 1 l at a time. Six membrane filters per month per location were stored at − 80 °C for later DNA extraction.

### Histopathology

Tissue histology was used as the secondary tool after PCR, to screen a subset of animals to estimate the severity of, and potential immune responses to, any *Hematodinium* (e.g. melanisation reactions, haemocyte aggregation). Three gills and three portions (*c.*0.5 cm^3^) of the hepatopancreas/gonad were excised and fixed in Davidson’s seawater fixative [29] for 24 h prior to their storage in 70% ethanol. Samples were dehydrated in a graded series of ethanol, transferred to Histoclear/Histochoice (Sigma-Aldrich, Dorset, UK) and infiltrated with molten wax using a Shandon™ automated tissue processor (Thermo Fisher Scientific, Altrincham, UK) prior to embedding. Blocks were cut at 5–7 µm thickness using an RM2245 microtome (Leica, Wetzlar, Germany). Sections were mounted on glass slides using albumin-glycerol fixative and stained with Cole’s haematoxylin and eosin. Stained slides were viewed and imaged using an Olympus BX41 microscope. Images were adjusted for colour balance and contrast only. Gills and hepatopancreas found to be positive for *Hematodinium* sp. *via* PCR were graded 0–4 for infection severity according to the criteria of Smith et al. [6] (0 signifies subclinical infections, undetected by histology but positive by PCR). The subset screened consisted of all *Hematodinium*-positive samples using PCR, plus an equal number of control (apparently disease-free) crabs of the same size and sex.

### DNA extraction and quantification

Crab DNA was extracted from 100 µl of thawed haemolymph using Qiagen Blood and Tissue Kits (Qiagen, Hilden, Germany) and water eDNA was extracted from each thawed filter membrane using a Qiagen DNeasy PowerWater Kit, both following the manufacturer’s instructions. Extracted DNA was quantified using a Qubit^®^ dsDNA High Sensitivity Assay Kit and Qubit^®^ Fluorometer (Invitrogen, California, USA). Following quantification, water eDNA generated from filter membranes of the same replicate site/same month/same location were pooled in equimolar concentrations to give 3 samples per location, per month to be used in downstream analysis.

### PCR and sequencing conditions

All PCR reactions were carried out in 25 μl total reaction volumes using 2× Master Mix (New England Biolabs Inc., Ipswitch, USA), oligonucleotide primers synthesized by Eurofins (Ebersberg, Germany), 1 μl of genomic DNA (*c*.50–200 ng/μl) and performed on a T100 PCR thermal cycler (BioRad Laboratories Inc., Hemel Hempstead, UK). Products derived from PCR were visualized on a 2% agarose/TBE gel with GreenSafe premium nucleic acid stain (NZYTech, Lisboa, Portugal). For primary diagnostics, general *Hematodinium* primers targeting a highly variable *18S* rRNA gene region (Hemat-F-1487 and Hemat-R-1654, Table 1) were used to verify the presence of any *Hematodinium* in the extracted DNA. If samples contained a positive signal for *Hematodinium* in the first instance, a second round of PCR was performed with *Hematodinium* spp.-specific primers with a larger fragment suitable for sequencing (18SF2 and Hem3R, Table 1). Finally, if the second set of primers did not amplify the fragment for sequencing, samples were interrogated further with alternative *Hematodinium* spp.-specific primers, 18SF2 and ITSR1 (Table 1). Positive samples were re-amplified and purified using HT ExoSAP-IT™ Fast high-throughput PCR product cleanup (Thermo Fisher Scientific, Altrincham, UK) in preparation for target sequencing. Amplicons were identified by DNA Sanger sequencing using both forward and reverse primers synthesised by Source BioScience (Nottingham, UK) and Eurofins.

**Table 1:** Forward and reverse primer sequences used for the amplification of *Hematodinium* by PCR. Each PCR run included initial denaturation and final extension steps, according to the first and final temperatures, respectively, noted in the thermocycler settings

Primers				Thermocycler settings			Amplicon size (bp)	References
Direction	Name	Sequence (5′–3′)	Final concentration (µM)	Temperature (°C)	Time	No. of cycles		
Forward	Hemat-F-1487	CCTGGCTCGATAGAGTTG	0.5	94	10 min	30	187	[57]
Reverse	Hemat-R- 1654	GGCTGCCGTCCGAATTATTCAC		94	15 s			
				54	15 s			
				72	30 s			
				72	10 min			
Forward	18SF2	CAGTTTCTGGAAGTGGCAGCTG	1	94	1 min	35	480	[58, 59]
Reverse	Hem3R	TAACCCGAGCCGAGGCATTCA		94	1 min			
				58	1 min			
				72	1 min			
				72	10 min			
Forward	18SF2	CAGTTTCTGGAAGTGGCAGCTG	0.5	94	1 min	35	380	[58]
Reverse	ITS R1	GAAGGGAAGGGGAGAAGAAGC		94	30 s			
				57	1 min			
				72	1 min			
				72	7 min			

### Phylogenetic analyses

Consensus sequences were constructed from clipped sequences using the CAP contig assembly extension in BioEdit sequence alignment editor [30]. Reference sequences of the respective region from *H. perezi* and *Hematodinium* sp. recovered from a broad range of crustacean hosts were sourced from GenBank at NCBI [31]: *Callinectes sapidus*, *Chionoecetes angulatus*, *C. bairdi*, *C. opilio*, *C. tanneri*, *Cancer pagurus*, *Carcinus maenas*, *Exopalaemon carinicauda*, *Hyas coarctatus*, *H. araneus*, *Liocarcinus depurator*, *Lithodes couesi*, *Munida rugosa*, *Nephrops norvegicus*, *Pagurus bernhardus*, *P. prideaux*, *Penaeus monodon*, *Portunus trituberculatus* and *Scylla paramamosain*. Sequences from *Amoebophyra* species (GenBank: HM483395, HQ658161, HM483394 and MK681270) were used as an outgroup for the trees. Multiple sequence alignments were performed in CLUSTAL X v.2 [32]. Evolutionary analyses and reconstructions were carried out in MEGA X [33] using the maximum likelihood routine based on the Tamura-Nei model. A consensus tree with the highest log likelihood value (− 250.10) from 1000 bootstrap re-samplings was annotated using iTOL software [34]. All sequences have been deposited in the GenBank database under the accession numbers MN057783–MN057918 for crab DNA and MN049783–MN049789 for water eDNA (see Additional file 1: Table S4).

### Statistical analyses

Sample size calculations using an alpha value of 0.05 and desired power > 80% indicated a minimum of 38 (1-sided test) up to 48 (2-sided test) crabs were needed based on an *a priori* prediction of 15% *Hematodinium* prevalence in the *C. maenas* population (in line with findings of Smith et al. [6] when screening *C. pagurus*).

Binomial logistic regression models with Logit link functions (following Bernoulli distributions) were used (MASS library) to determine whether specific predictor variables had a significant effect on the probability of finding crabs testing positive for *Hematodinium* presence in the crab populations sampled. All logistic models were run in RStudio v.1.1.463 using R v.3.5.1. The information theoretic approach was used for model selection and assessment of performance [35]. Initial models are herein referred to as the full models. Once selected, each non-significant predictor variable from the full models was sequentially removed using the drop1 function to produce final models with increased predictive power, herein referred to as the reduced models. The drop1 function compares the initial full model with the same model, minus the least significant predictor variable. If the reduced model is significantly different from the initial full model (in the case of binomial response variables, a Chi-square test is used to compare the residual sum of squares of both models), then the removed predictor variable is kept out of the new, reduced model. This process continues hierarchically until a final reduced model is produced [36]. Full models included the input variables: season [winter (Dec ’17, Jan ’18, Feb ‘18), spring (Mar ’18, Apr ’18, May ’18), summer (Jun ’18, Jul ’18, Aug ’18), autumn (Sept ’18, Oct ’18, Nov ’17)], CW (continuous number), sex (male or female), colour (green, yellow or orange), pigment loss (0 or 1), haemolymph opacity (milky or clear), fouling (presence of epibionts, 0 or 1) and limb loss (0 or 1). Location (Pier or Dock) was also used in the first model before sites were separated. Graphics were produced using GraphPad Prism v.8.00 for Windows.

## Results

### General population observations

**Fig. 2:**
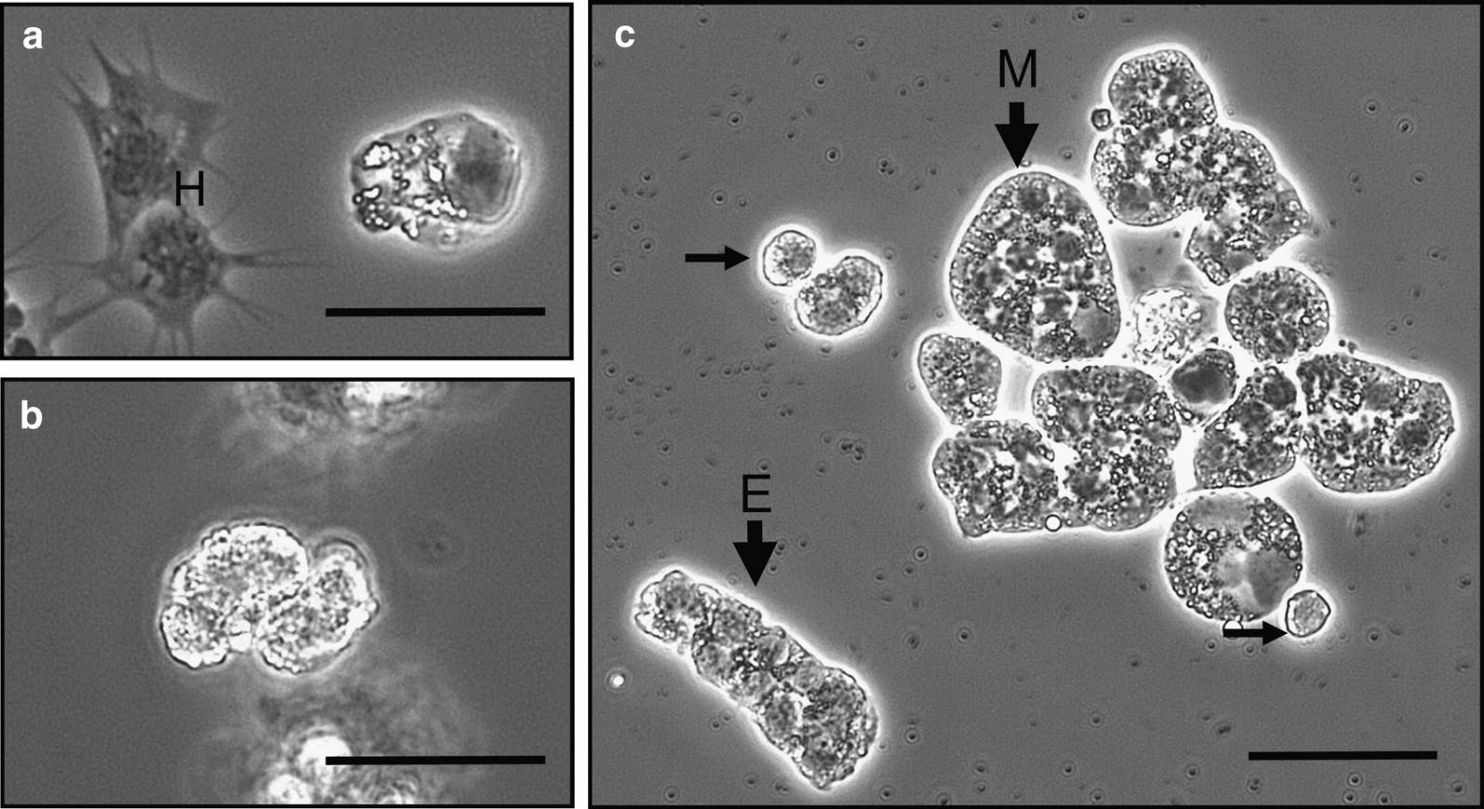
Identification of *Hematodinium* in fresh haemolymph preparations using phase contrast microscopy. Parasites were identified by their lack of attachment and spreading to slides (*cf*. the haemocytes, H) and their highly refractile nature and irregular sizes and shapes. Parasites were found singly (**a**, **b**) particularly in low severity infections or in clumps (**c**) in those crabs with high intensity infections. Note the variation in size and shape of the parasites in panel **c** with elongate (E), multinucleate (M) and small (unlabelled arrows) forms. *Scale-bars*: 25 µm

Overall, 1191 crabs were sampled across the yearlong survey, 603 from the Dock and 588 from the Pier. Of these crabs combined, 9.4% were *Hematodinium*-positive using the haemolymph screen alone (Fig. 2a–c), whereas 13.6% were *Hematodinium-*positive *via* PCR, suggesting a larger sub-clinical or carrier presence in the population. The initial screening for the presence of *Hematodinium* in haemolymph was based on their morphological differences to the host haemocytes. Various forms of *Hematodinium* were non-adherent (unlike the haemocytes that attach and spread to the slides) and irregular in shape and size with variable refractivity (Fig. 2a–c). Herein, when referring to the presence of *Hematodinium*, we refer to the occurrence *via* PCR alone unless otherwise stated.

**Fig. 3:**
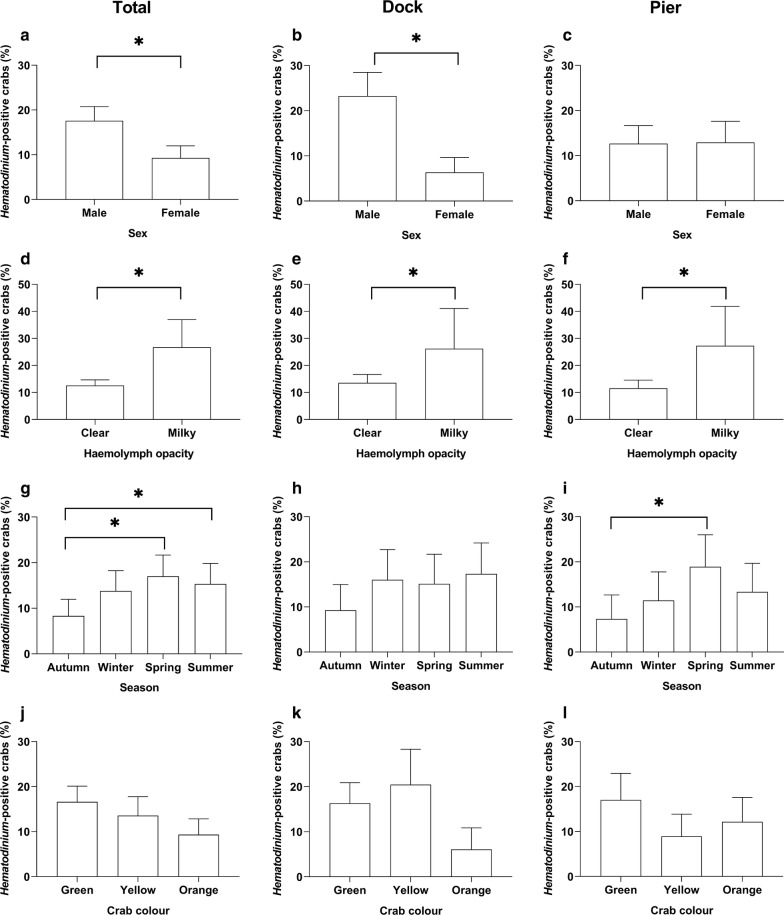
Percentage of *Hematodinium* sp. in *Carcinus maenas* per location: total population, Dock and Pier, according to predictor variables sex (**a**–**c**), haemolymph opacity (**d**–**f**), season (**g**–**i**) and crab colour (**j**–**l**). Values represent mean + 95% CI, asterisk denotes significant difference (*P* ≤ 0.05)

**Fig. 4:**
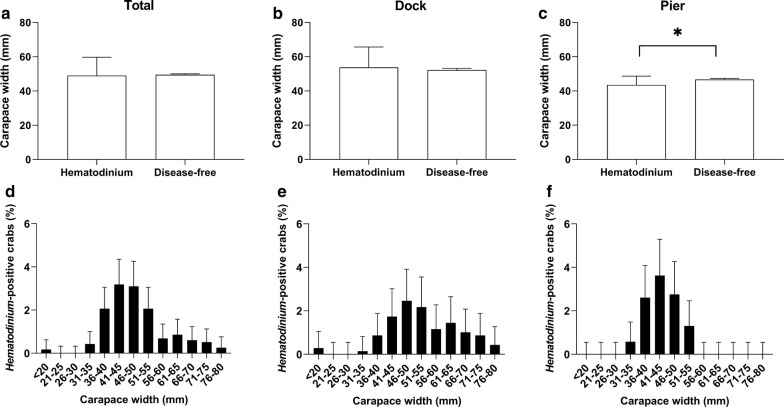
Carapace width (mm) of *C. maenas* presenting *Hematodinium* and those ‘*Hematodinium*-free’ per location: total population (**a**), Dock (**b**) and Pier (**c**). Also shown, size distribution in 5 mm size groups of *C. maenas* presenting *Hematodinium* per location: total population (**d**), Dock (**e**) and Pier (**f**). Values represent mean + 95% CI, asterisk denotes significant difference (*P* ≤ 0.05)

Model 1 combined the data from both locations, and using the presence of *Hematodinium* as the response variable, revealed that season, sex and haemolymph opacity were significant factors associated with the presence of the parasite (Table 2, Model 1). Of the male crabs, 17.6% were *Hematodinium*-positive whereas just 9.3% of the females presented the disease, making males nearly twice as likely to become infected (Fig. 3a–c). In terms of haemolymph opacity, 26.7% of crabs that displayed milky or cloudy white haemolymph were *Hematodinium*-positive whereas just 12.6% of those with clear or ‘normal’ haemolymph were diseased (Fig. 3d–f). In terms of seasonality, those crabs found in spring (March-May) and summer (June–August) were significantly more likely to be infected by *Hematodinium* than those found in the autumn/winter (November–January) (17, 15.3, 8.3%, respectively; Fig. 3g–i). Size (carapace width), crab colour (Fig. 3j–l), pigment loss, fouling (presence of epibionts), limb loss and location did not have a significant effect (Fig. 4a, d; Additional file 1: Table S1, Model S1).

**Table 2:** Binomial logistic regression models (reduced from the full models, Additional file 1: Table S1) testing the effects of biometric and environmental predictor variables on the overall presence of *Hematodinium* in the population. Models separated by location: Model 1, total population; Model 2, Dock; Model 3, Pier

Model	Predictor variable	Estimate (slope)	SE	*P*-value
Model 1				
Hemat ~ Season + Sex	Season (spring)	0.8137	0.2628	0.00196**
+ HemoCol	Season (summer)	0.7437	0.2670	0.00535**
*df* = 1188	Season (winter)	0.4678	0.2740	0.08776
AIC: 914.72	Sex (male)	0.7940	0.1828	1.4e−05***
	HemoCol (milky)	1.1187	0.2716	3.8e−05***
Model 2				
HematDock ~ Sex	Sex (male)	1.4790	0.2918	7.4e−07***
+ Colour + HemoCol	Colour (orange)	− 0.5830	0.3827	0.1277
*df* = 595	Colour (yellow)	0.3667	0.2808	0.1915
AIC: 450.92	HemoCol (milky)	1.1323	0.4070	0.0054**
Model 3				
HematPier ~ Season	Season (spring)	0.99428	0.38751	0.010293*
+ CW + HemoCol	Season (summer)	0.75876	0.40444	0.060645
*df* = 586	Season (winter)	0.35552	0.42248	0.400067
AIC: 430.7	CW	− 0.06889	0.02065	0.000848***
	HemoCol (milky)	1.17749	0.38636	0.002306**

*Statistically significant *P ≤ 0.05, **P ≤ 0.01, ***P ≤ 0.001

*Abbreviation*: SE, standard error

### Presence of *Hematodinium* in crabs by location

To further explore the possible relationship between external factors and the presence of *Hematodinium*, the data were separated and analysed between the two locations (i.e. Dock *vs* Pier). In the Dock, 14.4% of crabs surveyed presented *Hematodinium*. Using the presence of *Hematodinium* in the Dock as the response variable (Model 2) revealed that sex and haemolymph opacity were significant factors associated with the presence of *Hematodinium* (Table 2). Of the male crabs in the Dock, 23.3% presented *Hematodinium* whereas 6.4% of females were diseased, making males more than three times as likely to become infected (Fig. 3b). In terms of haemolymph opacity, 26.2% of crabs that displayed milky or cloudy white haemolymph were *Hematodinium*-positive whereas 13.5% of those with clear or ‘normal’ haemolymph were diseased (Fig. 3e). Season, size (CW), pigment loss, fouling (presence of epibionts), limb loss and location did not have a significant effect (Additional file 1: Table S2, Model S2). The drop1 function deemed crab colour significant enough to be kept in the in the final (reduced) model; however, it had no significant final effect on the presence of *Hematodinium* in crabs from the Dock (Model 2, Table 2, Fig. 3j).

In the Pier location, 12.8% of crabs surveyed presented *Hematodinium*. Using the presence of *Hematodinium* in the Pier location as the response variable (Model 3), revealed that season, size (CW) and haemolymph opacity were significant factors associated with the presence of *Hematodinium* (Table 2, Model 3). Those crabs found in the Pier in spring (March–May) were significantly more likely to have *Hematodinium* than those found in autumn (September–November) and highest overall (18.9 and 7.3%, respectively; Fig. 3i). In terms of size, smaller crabs were significantly more likely to display *Hematodinium* compared to parasite-free crabs (mean ± SD: 43.50 ± 5.14 *vs* 46.68 ± 7.00 mm, respectively; Fig. 4c, f). In terms of haemolymph opacity, 27.3% of crabs that displayed milky or cloudy haemolymph were *Hematodinium-*positive, whereas just 11.6% of those with clear, ‘normal’ haemolymph were diseased (Fig. 3f). Sex, crab colour, pigment loss, fouling (presence of epibionts), limb loss and location did not have a significant effect (Additional file 1: Table S3, Model S3).

### Severity of *Hematodinium* in infected crabs

**Fig. 5:**
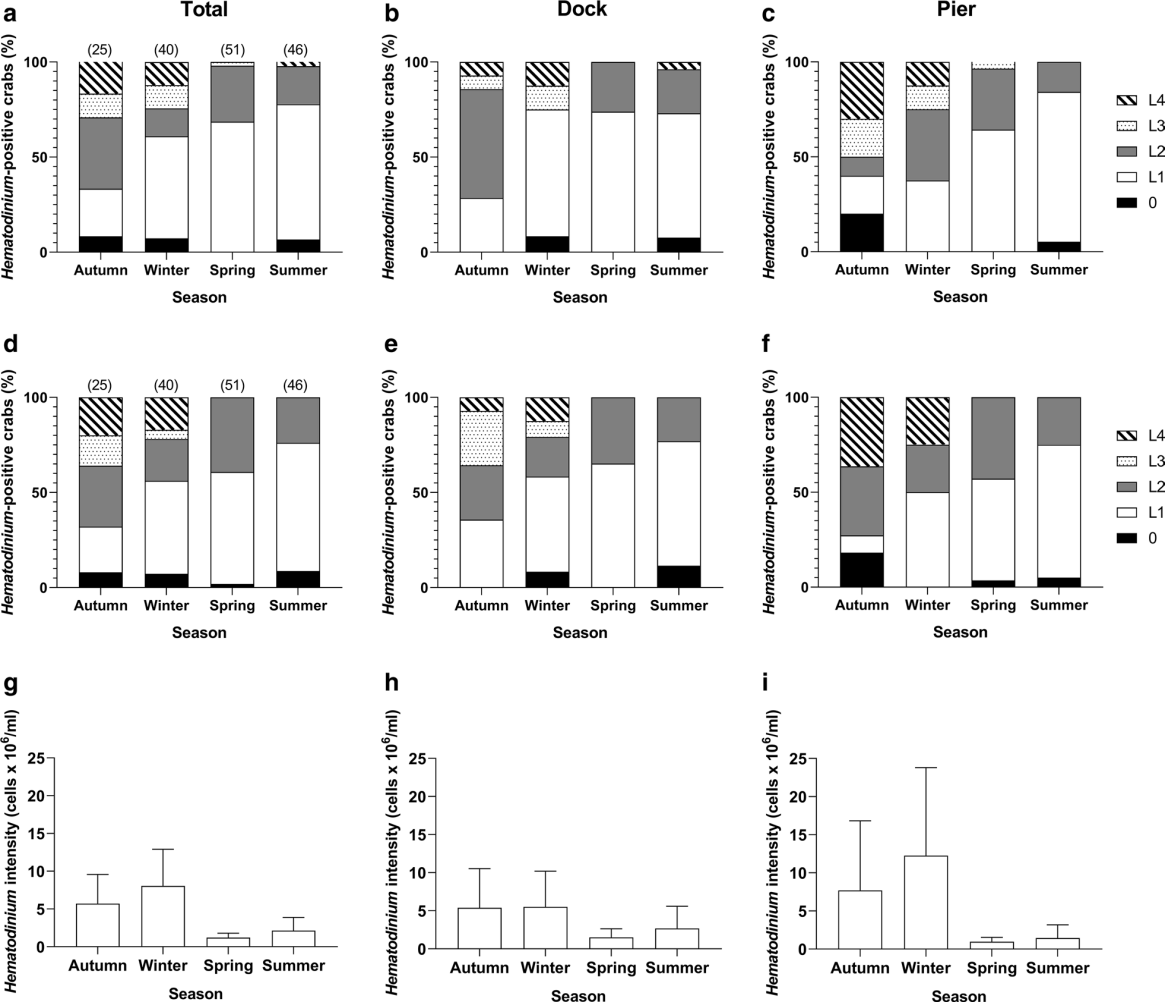
Temporal changes in the severity of *Hematodinium* infection of *C. maenas* in gill (**a**–**c**) and hepatopancreas (**d**–**f**) histopathology. *Hematodinium* presence was first determined *via* PCR, and for all positives, severity of infection was determined using histological analysis (L1 being the lowest, L4 being the highest; see Smith et al. [6] for grading criteria; zero signifies subclinical infections, undetected by histology but positive by PCR). Values in parentheses are the total number of positive crabs identified by PCR in each sample. **g**–**i** Parasites/ml haemolymph as a marker of severity, using crabs *Hematodinium-*positive *via* haemolymph preparations only (i.e. clinical infections)

Although the presence of *Hematodinium* in shore crabs was lowest in the autumn to winter months (September–February), high severity infections (levels L3 and L4) were more prevalent in histological examination of both the gills and the hepatopancreas during these seasons (Fig. 5). Low severity infections (L1) were more prevalent in spring (March–May) and summer (June–August) months (Fig. 5). These data indicate that severity and prevalence of *Hematodinium* have opposite seasonal patterns with high severity and low prevalence in autumn-winter and low severity and higher prevalence in spring-summer.

**Fig. 6:**
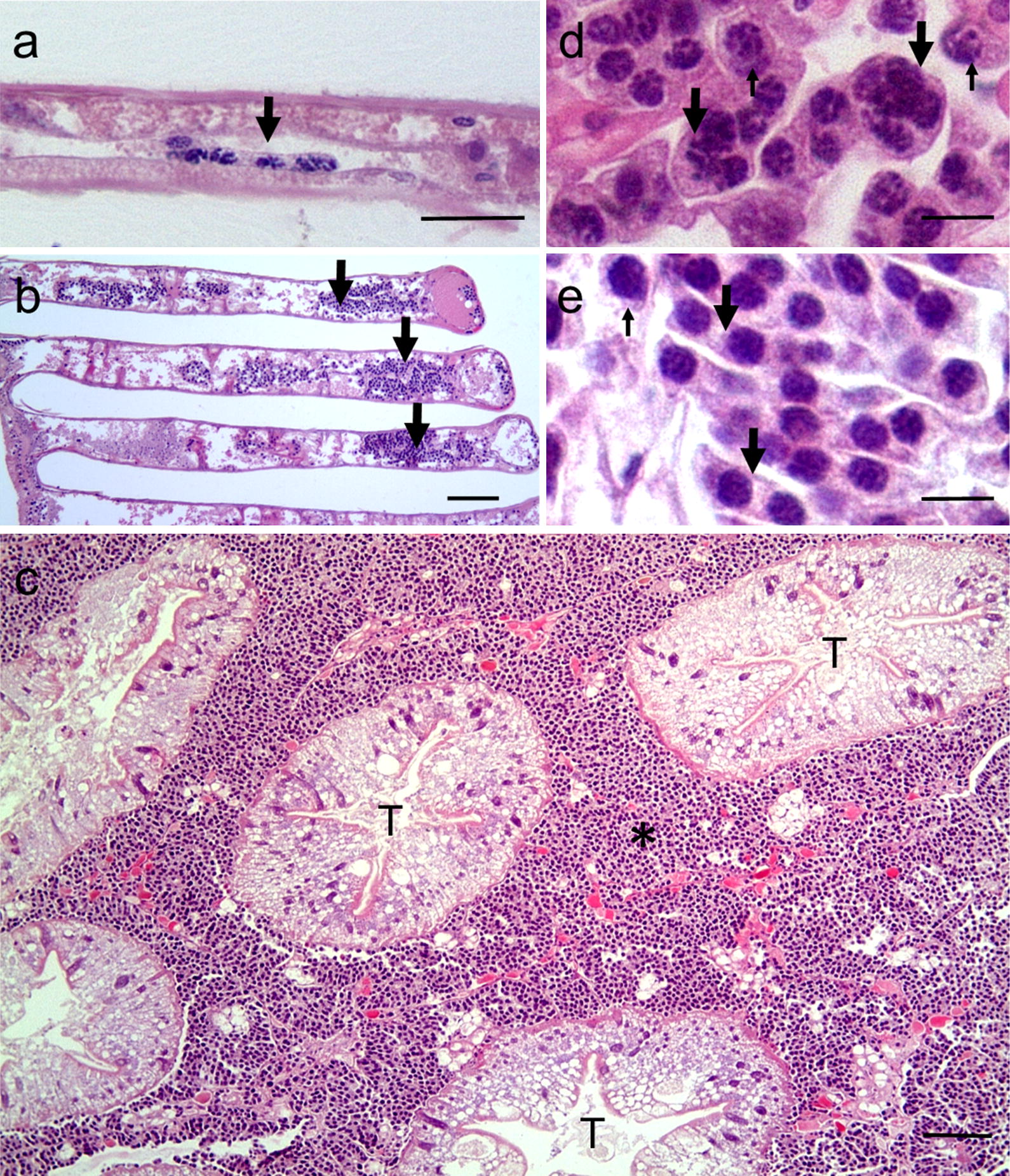
Histopathology of *Hematodinium* infections in *C. maenas*. **a** Multinucleate elongate form of the parasite (arrow) seen attached to the wall of haemolymph channel in gill lamella in a low severity (grade L1) infection. **b** Low power micrograph of gill lamellae from a crab with high severity (grade L4) infection showing large numbers of parasites (arrows). **c** Low power micrograph of the hepatopancreas from a crab with high severity infection. The intertubular space (*) is replete with different forms of *Hematodinium*. **d**, **e** High power micrographs showing the diversity of forms of the parasite including rounded (small arrows), multinucleate rounded and elongate forms (large arrows). *Abbreviation*: T, tubules. *Scale-bars*: **a**, 25 µm; **b**, **c**, 100 µm; **d**, **e**, 10 µm

Histological examination revealed changes in the morphology of these parasites depending on the severity of infection. For example, in low severity infections (L1) the *Hematodinium* were often elongate and multinucleate forms attached to host tissues such as the gills (Fig. 6a), together with rounded forms apparently free in circulation. In high severity infections (L3–4), gill lamellae were filled with *Hematodinium* (Fig. 6b) and intertubular spaces in the hepatopancreas were swollen and replete with these parasites (Fig. 6c). The *Hematodinium* in these spaces in both gills and hepatopancreas were a mix of rounded, elongate and multinucleate forms (Fig. 6d, e) similar to those seen in the haemolymph preparations examined using phase contrast microscopy (Fig. 2a–c). There was no evidence of any direct host response to the presence of *Hematodinium* in the tissues such as encapsulation/nodule formation [37]. Where encapsulation of damaged or necrotic host tissues did occur, i.e. in the tubules of the hepatopancreas, these events were independent of the presence of these parasites and the various forms of *Hematodinium* were not seen within the haemocyte sheaths surrounding damaged tissues (not shown). In *Hematodinium*-infected crabs, there was no evidence that tissue damage was caused by the presence of *Hematodinium* alone. No gross differences in the histopathology of *Hematodinium* infections were seen between crabs collected from either the Docks or the Pier.

### Presence of *Hematodinium* in water samples

Of the 69 water samples (36 for Pier, 33 for Dock) screened using the primary Hemat-F-1487/Hemat-R-1654 oligonucleotides, no water sample from the Dock location was positive. In the Pier location, the water samples from the months of November and December, across all 3 replicates, plus 1 replicate from August were positive for *Hematodinium*. From these positive samples, none amplified successfully using the subsequent 18SF2/Hem3R or 18SF2/ITSR1 oligonucleotides and so were sequenced using the primary Hemat-F-1487/Hemat-R-1654 oligonucleotides and deposited in the GenBank database under the accession numbers MN049783–MN049789 (Additional file 1: Table S4).

### Phylogenetic analyses

**Fig. 7:**
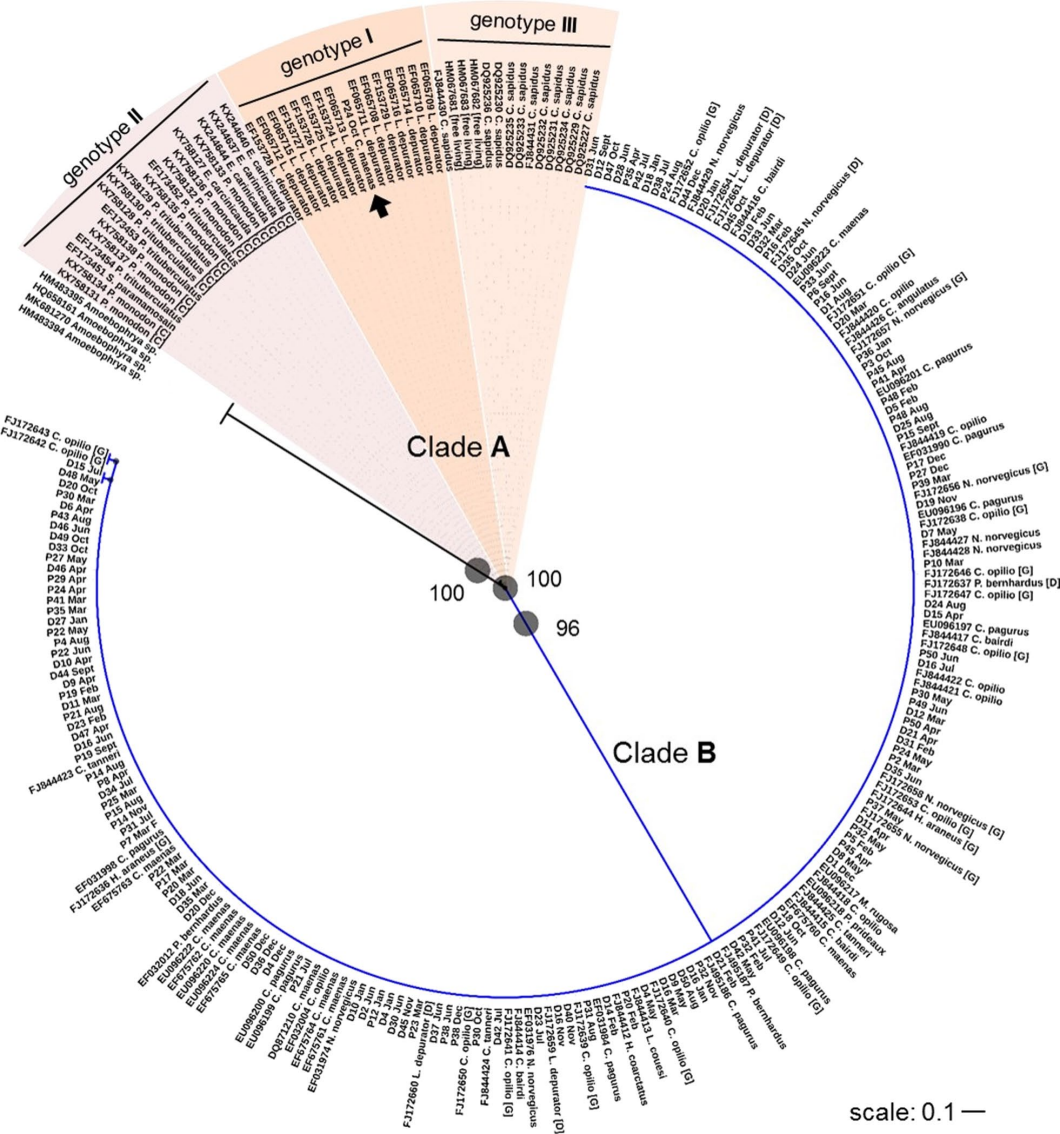
Consensus phylogram of the ITS1 and partial *18S* rRNA gene regions from *Hematodinium*-infected crustaceans (maximum likelihood estimation, 1000 bootstrap replicates). Genomic DNA was isolated from the haemolymph of infected shore crabs across two locations (prefix P, Pier location; prefix D, Dock location) in Swansea Bay, UK (GenBank: MN057783–MN057918, see Additional file 1: Table S4 for individual numbers), and probed *via* PCR for *Hematodinium* diversity. Reference nucleotide sequences for *Hematodinium* from various crustacean hosts (see methods for full list) were retrieved from GenBank. The spheres/numbers at the nodes indicate bootstrap support (%) received for each partition. The tree was rooted using the corresponding region from *Amoebophyra* species (GenBank: HM483395, HQ658161, HM483394, MK681270). Postfix: C, China; D, Denmark; G, Greenland

Of the 162 *Hematodinium-*positive crab samples using the Hemat-F-1487/Hemat-R-1654 oligonucleotides, 149 were re-amplified successfully for sequencing using the 18SF2/Hem3R and 12 with the 18SF2/ITSR1 oligonucleotides. One sample (Pier 40 April) did not amplify successfully using the 18SF2/Hem3R or 18SF2/ITSR1 oligonucleotides and was instead sequenced with the Hemat-F-1487/Hemat-R-1654 oligonucleotides. Following quality control, 136 of these sequences (of the ITS1 and partial *18S* rRNA gene regions of *Hematodinium*) were combined with 126 reference sequences for evolutionary analyses (Fig. 7) and deposited in the GenBank database under the accession numbers MN057783–MN057918 (Additional file 1: Table S4). A single sequence, namely Pier 24 October, shared considerable similarity (490 bp, 100% coverage, 98.2% identity) to a *H. perezi* clone from the harbour crab *L. depurator* (GenBank: EF065708) by Small et al. [38]. The remaining 135 sequences shared high similarity (> 95% coverage and identity) with the so-called *Hematodinium* sp. clones retrieved from a plethora of hosts, including shore crabs (*Carcinus maenas*), edible crabs (*Cancer pagurus*), tanner crabs (*Chionoecetes* spp.) and langoustines (*N. norvegicus*). The topology of the consensus phylogram revealed two distinct, highly supported, clades of *Hematodinium* A and B (Fig. 7). Clade A consists entirely of *H. perezi* and forms three clusters with respect to established genotypes (I, *L. depurator*; II, South-East Asia; III, *C. sapidus*), which is in good agreement with several previous assessments [38–41]. Sequences from *Hematodinium*-positive crabs across both locations (Pier and Dock) and every month of the year-long survey are distributed within Clade B, thereby suggesting that the parasite most likely infecting *C. maenas* in our two locations is the generalist *Hematodinium* sp.

## Discussion

The parasitic dinoflagellate *Hematodinium* is present in common shore crabs across at least two locations in Swansea Bay, UK (the Prince of Wales Dock and Mumbles Pier), with both the general *Hematodinium* sp. and *H. perezi* detected. Both locations sampled showed a seasonal trend of *Hematodinium* presence, with high prevalence but low severity (i.e. low parasite load) of infection in the haemolymph and gill/hepatopancreas histopathology in spring to summer. In the autumn months, the number of crabs found to harbour *Hematodinium* was significantly lower but these individuals had higher severity infections. This gross *Hematodinium* burden in autumn/winter crabs is accompanied by clear signals of *Hematodinium* eDNA in the surrounding waters (in November and December), indicating that infectious morphs of the parasite are liberated to target other hosts at this time. Aside from seasonality, haemolymph opacity, sex and size were also associated with the presence of the parasite. In terms of phylogeny, the vast majority of the *Hematodinium* sp. found in this study (> 99%) reside in Clade B, alongside other generalist *Hematodinium* sequences.

The role of seasonality in relation to *Hematodinium* presence has been noted in studies of many host species [6, 10, 12, 42–50]. Seasonal prevalence of the parasite also seems to be host specific, mostly related to location, and therefore temperature and salinity. We found that *Hematodinium* prevalence is high with a low infection intensity in the spring/summer months. Chualáin et al. [42] noted that infection intensity rather than prevalence played an important role in the presence of *Hematodinium*. In that study, it was found that intensity of *Hematodinium* infection was significantly higher, with peaks occurring in late autumn/early winter months. Smith et al. [6] recorded similar patterns in *C. pagurus* in two locations in South Wales (including Mumbles Pier, as in the present study) with high numbers of animals infected in the spring to summer but with low severity. Instead, in November, fewer crabs were infected but these animals had hefty parasite loads in their haemolymph and other tissues. These results suggest that seawater temperature or an environment-linked process could be a key factor in triggering the final stages of infection. The apparent presence of *Hematodinium* in all seawater eDNA samples in November and December in the present study is further evidence for this hypothesis. After peak *Hematodinium* prevalence in spring/summer, development of the parasite within host haemolymph and tissue could lead to high severity in a small number of surviving crabs by autumn and winter. The presence of *Hematodinium* in seawater eDNA samples is probably from moribund individuals releasing infective stage dinospores into the water, in turn causing the low severity infections seen the following spring (described above). *Hematodinium* sp. have also been found in environmental samples (seawater and sediment) in Maryland and Virginia coastal bay ecosystems in the USA, whereby the ‘free-living’ *Hematodinium* sp. occurred in the ecosystem earlier than peak infection presence in the crabs, a similar observation to ours [18].

In the present study, sex played a role in the presence of *Hematodinium* in the Dock location only. These results are in line with previous work, whereby male *C. maenas* in the Clyde Sea, Scotland were found with higher levels of *Hematodinium via* PCR (e.g. [10]). Additionally, male *C*. *pagurus* in the north and southeast of Ireland were found with higher levels of *Hematodinium* [42]. Whilst smaller crabs were significantly more likely to display *Hematodinium* in the Pier location only, this phenomenon has also been observed in *N. norvegicus*, whereby infection prevalence was highest in smaller individuals [4]. This pattern is common in many species. For example, medium size and juvenile *C. sapidus* (≤ 30 mm CW) have the highest infection prevalence [51]. It has been suggested that since smaller crustaceans moult more frequently, there will be a greater parasite prevalence as moulting can leave the crustacean vulnerable to pathogen entry [4, 10, 52]. It must be noted that although overall and in the Pier location higher numbers of most recently-moulted (green) crabs were *Hematodinium-*positive, this difference was not deemed significant in the final models. The absence of a size-related pattern in the Dock location could be because it is a semi-closed location and is unaffected by tidal height unlike the Pier location. Dock crabs may be more settled and less likely to move as in the ‘open’ Pier location.

Phylogenetic reconstructions demonstrated clearly that there was little difference in the ecotype diversity of *Hematodinium* sequences between location (Pier and Dock) or month. Most sequences were distributed within Clade B, with a single sequence in the *H. perezi*-dominated Clade A. This suggests that the parasite infecting *C. maenas* across both locations is most likely the generalist *Hematodinium* sp. The taxonomic diversity of *Hematodinium* spp. has been discussed at length in the literature. Small [53] reviewed the global diversity and distribution of these parasites and most notably, Hamilton et al. [54] compared the genotypic variability of *Hematodinium* from North Atlantic hosts and presented three clades, corresponding to host species, rather than to geographical location. Jensen et al. [39] presented evidence of two clades of *Hematodinium* in the northern hemisphere: one clade (A) isolated from *C. sapidus* and *L. depurator* and the other clade (B) found in all other host species from both the North Atlantic and Pacific Oceans. Clade A was affiliated with the type-species *H. perezi* identified by Small et al. [38] and three distinct genotypes (I, II, III): Genotype I in the English Channel; Genotype II off the east coast of China; and Genotype III along the east coast of the USA [38, 40]. These genotypes have also been referred to as ‘Clades’ [41]. By combining our sequence data with 126 references from GenBank, we provide strong evidence in agreement with previous studies that there are two broad groups of disease-causing *Hematodinium*: namely (i) *H. perezi*, which can be separated by distinct host species and geographical locations; and (ii) *Hematodinium* sp., which is pervasive.

The edible (brown) crab is worth around GBP 50 million per year to the UK and Ireland, and lives alongside shore crabs when in the intertidal zone [55]. Previous reports of *Hematodinium* presence in pre-recruit edible crabs in the Bristol Channel indicate up to 30% of individuals are infected [6]. Our data demonstrate clearly that shore crabs in this commercially important region can facilitate *Hematodinium* persistence. As discussed, severity and temporal profiles are rather similar between the edible and shore crabs and the hypothesis that *C. maenas* may act as a vector for diseases in the commercially important *C. pagurus* remains a key finding of this study. Additionally, Hamilton et al. [10] found *Hematodinium* in seven crustacean species with prevalence reaching almost 60% in hermit crabs (*P. bernhardus*) and 30% in *C. pagurus* and *N. puber*, with prevalence in *Munida rugosa* and *Pagurus bernhardus*, peaking in April (much like *C. maenas* in the present study). This is pertinent as transfer of *Hematodinium* between species most likely occurs *via* predation of infected animals, and hermit crabs are common in the diet of larger predatory crabs. Lohan et al. [56] also examined *c.*1800 crustaceans along the Delmarva Peninsula (Virginia, USA) over a two-year period and found *Hematodinium* in five hosts additional to the American blue crab, *C. sapidus*. Sequencing of the ITS1 region demonstrated clearly that the same *Hematodinium* found in the commercially sensitive blue crabs is present in taxonomically diverse hosts, including an amphipod (*Caprella geometrica*).

## Conclusions

The seasonal trend of *Hematodinium* sp. presence in host crabs, as well eDNA signals, confirm a whole parasite life-cycle in Swansea Bay, enabling us to ‘track’ it in the water column and into the host. The association of *Hematodinium* sp. presence with both crab sex and size indicates a relationship with moulting, also noted by other studies. Encountering *Hematodinium* at relatively high percentages across two small populations gives an insight into reservoirs of crustacean diseases in the aquatic environment, with implications for commercially important species sharing the same habitat.

## Supplementary information

**Additional file 1: Table S1.** Full model used in order to predict response variable of presence of *Hematodinium* sp. before reduction. Asterisk denotes significance (*P* ≤ 0.05). **Table S2.** Full model used in order to predict response variable of presence of *Hematodinium* sp. in the Dock location before reduction. Asterisk denotes significance (*P* ≤ 0.05). **Table S3.** Full model used in order to predict response variable of presence of *Hematodinium* sp. in the Pier location before reduction. Asterisk denotes significance (*P* ≤ 0.05). **Table S4.** Accession numbers, deposited in GenBank, and corresponding sampling numbers for all *Hematodinium-*positive animals successfully sequenced from study, and used in the phylogenetic tree (Fig. 7).

## Data Availability

All data generated or analysed during this study are included in this published article and its additional files. All newly generated DNA sequences have been submitted to the GenBank database under the accession numbers MN057783–MN057918 for crab DNA and MN049783–MN049789 for water eDNA.

## Abbreviations

CW: carapace width
NCBI: National Center for Biotechnology Information
PCR: polymerase chain reaction
rRNA: ribosomal ribonucleic acid
TBE: tris-borate-ethylenediaminetetraacetic acid
