# Supplementary material for: Spatial and temporal disease dynamics of the parasite Hematodinium sp. in shore crabs, Carcinus maenas
Source: Parasit Vectors. 2019 Oct 11;12:472. doi: 10.1186/s13071-019-3727-x (PMC6790014; doi:10.1186/s13071-019-3727-x)
Supplement: Supplementary file 1 — Additional file 1: Table S1. Full model used in order to predict response variable of presence of Hematodinium sp. before reduction. Asterisk denotes significance (P ≤ 0.05). Table S2. Full model used in order to predict response variable of presence of Hematodinium sp. in the Dock location before reduction. Asterisk denotes significance (P ≤ 0.05). Table S3. Full model used in order to predict response variable of presence of Hematodinium sp. in the Pier location before reduction. Asterisk denotes significance (P ≤ 0.05). Table S4. Accession numbers, deposited in GenBank, and corresponding sampling numbers for all Hematodinium-positive animals successfully sequenced from study, and used in the phylogenetic tree (Fig. 7). [file 13071_2019_3727_MOESM1_ESM.docx]

**Additional file 1**

**Table S1.** Full model used in order to predict response variable of presence of *Hematodinium* sp. before reduction. Asterisk denotes significance (α ≤ 0.05).

| **Model S1** | **Parameter** | **Estimate (slope)** | **p** |
| --- | --- | --- | --- |
| Hemat ~ Location +Season + CW + Sex + Colour  + Pigment.Loss + HemoCol + Fouling + LimbLoss | LocationPier | -0.248423 | 0.215450 |
|  | SeasonSpring | 0.705248 | 0.011692 * |
| **AIC:** 886.6948 | SeasonSummer | 0.697531 | 0.012432 * |
|  | SeasonWinter | 0.295907 | 0.302607 |
|  | CW | -0.005133 | 0.609323 |
|  | SexMale | 0.785202 | 0.000158 * |
|  | ColourOrange | -0.304451 | 0.248155 |
|  | ColourYellow | -0.042942 | 0.850438 |
|  | Pigment.Loss | -0.046877 | 0.915221 |
|  | HemoColMilky | 1.157653 | 5.04e-05 * |
|  | Fouling | -0.127710 | 0.616808 |
|  | LimbLoss | 0.125309 | 0.552919 |

**Table S2.** Full model used in order to predict response variable of presence of *Hematodinium* sp. in the Dock location before reduction. Asterisk denotes significance (α ≤ 0.05).

| **Model S2** | **Parameter** | **Estimate (slope)** | **p** |
| --- | --- | --- | --- |
| HematDock ~ Season + CW + Sex + Colour +  Pigment.Loss + HemoCol + Fouling + LimbLoss | SeasonSpring | 0.535173 | 0.18820 |
|  | SeasonSummer | 0.632089 | 0.10296 |
| **AIC:** 453.2 | SeasonWinter | 0.507816 | 0.20959 |
|  | CW | 0.009259 | 0.42522 |
|  | SexMale | 1.429758 | 2.51e-06* |
|  | ColourOrange | -0.581189 | 0.15642 |
|  | ColourYellow | 0.479851 | 0.12175 |
|  | Pigment.Loss | -0.514771 | 0.63985 |
|  | HemoColMilky | 1.232745 | 0.00293* |
|  | Fouling | -0.171425 | 0.64322 |
|  | LimbLoss | 0.070010 | 0.81599 |

**Table S3.** Full model used in order to predict response variable of presence of *Hematodinium* sp. in the Pier location before reduction. Asterisk denotes significance (α ≤ 0.05).

| **Model S3** | **Parameter** | **Estimate (slope)** | **p** |
| --- | --- | --- | --- |
| HematPier ~ Season + CW + Sex + Colour +  Pigment.Loss + HemoCol + Fouling + LimbLoss | SeasonSummer | 0.61497 | 0.15010 |
|  | SeasonWinter | 0.10927 | 0.80414 |
| **AIC:** 416.62 | CW | -0.06861 | 0.00329* |
|  | SexMale | 0.14393 | 0.63280 |
|  | ColourOrange | -0.24604 | 0.51012 |
|  | ColourYellow | -0.46188 | 0.18379 |
|  | Pigment.Loss | -0.28232 | 0.56612 |
|  | HemoCol3Milky | 1.26419 | 0.00221* |
|  | Fouling | 0.21404 | 0.58669 |
|  | LimbLoss | 0.28358 | 0.35525 |

**Table S4.** Accession numbers, deposited in GenBank, and corresponding sampling numbers for all *Hematodinium*-positive animals successfully sequenced from study, and used in phylogenetic tree (Fig. 7).

| **GenBankID** | **Seq** | **Sample** | **Primers** | **Sample type** |
| --- | --- | --- | --- | --- |
| MN057783 | Seq1 | P14_Nov | Hematodinium (18SF2/Hem3R) | Haemolymph |
| MN057784 | Seq2 | P32_Nov | Hematodinium (18SF2/Hem3R) | Haemolymph |
| MN057785 | Seq3 | D16_Nov | Hematodinium (18SF2/Hem3R) | Haemolymph |
| MN057786 | Seq4 | D19_Nov | Hematodinium (18SF2/Hem3R) | Haemolymph |
| MN057787 | Seq5 | D40_Nov | Hematodinium (18SF2/Hem3R) | Haemolymph |
| MN057788 | Seq6 | D45_Nov | Hematodinium (18SF2/Hem3R) | Haemolymph |
| MN057789 | Seq7 | P17_Dec | Hematodinium (18SF2/Hem3R) | Haemolymph |
| MN057790 | Seq8 | P27_Dec | Hematodinium (18SF2/Hem3R) | Haemolymph |
| MN057791 | Seq9 | P38_Dec | Hematodinium (18SF2/ITS R1) | Haemolymph |
| MN057792 | Seq10 | D1_Dec | Hematodinium (18SF2/Hem3R) | Haemolymph |
| MN057793 | Seq11 | D4_Dec | Hematodinium (18SF2/Hem3R) | Haemolymph |
| MN057794 | Seq12 | D20_Dec | Hematodinium (18SF2/Hem3R) | Haemolymph |
| MN057795 | Seq13 | D36_Dec | Hematodinium (18SF2/Hem3R) | Haemolymph |
| MN057796 | Seq14 | D44_Dec | Hematodinium (18SF2/ITS R1) | Haemolymph |
| MN057797 | Seq15 | D50_Dec | Hematodinium (18SF2/Hem3R) | Haemolymph |
| MN057798 | Seq16 | P12_Jan | Hematodinium (18SF2/Hem3R) | Haemolymph |
| MN057799 | Seq17 | P33_Jan | Hematodinium (18SF2/Hem3R) | Haemolymph |
| MN057800 | Seq18 | P36_Jan | Hematodinium (18SF2/Hem3R) | Haemolymph |
| MN057801 | Seq19 | D4_Jan | Hematodinium (18SF2/Hem3R) | Haemolymph |
| MN057802 | Seq20 | D10_Jan | Hematodinium (18SF2/Hem3R) | Haemolymph |
| MN057803 | Seq21 | D16_Jan | Hematodinium (18SF2/Hem3R) | Haemolymph |
| MN057804 | Seq22 | D18_Jan | Hematodinium (18SF2/Hem3R) | Haemolymph |
| MN057805 | Seq23 | D20_Jan | Hematodinium (18SF2/Hem3R) | Haemolymph |
| MN057806 | Seq24 | D27_Jan | Hematodinium (18SF2/Hem3R) | Haemolymph |
| MN057807 | Seq25 | P5_Feb | Hematodinium (18SF2/Hem3R) | Haemolymph |
| MN057808 | Seq26 | P16_Feb | Hematodinium (18SF2/Hem3R) | Haemolymph |
| MN057809 | Seq27 | P19_Feb | Hematodinium (18SF2/Hem3R) | Haemolymph |
| MN057810 | Seq28 | P20_Feb | Hematodinium (18SF2/Hem3R) | Haemolymph |
| MN057811 | Seq29 | P32_Feb | Hematodinium (18SF2/Hem3R) | Haemolymph |
| MN057812 | Seq30 | P48_Feb | Hematodinium (18SF2/Hem3R) | Haemolymph |
| MN057813 | Seq31 | D5_Feb | Hematodinium (18SF2/ITS R1) | Haemolymph |
| MN057814 | Seq32 | D10_Feb | Hematodinium (18SF2/Hem3R) | Haemolymph |
| MN057815 | Seq33 | D14_Feb | Hematodinium (18SF2/Hem3R) | Haemolymph |
| MN057816 | Seq34 | D21_Feb | Hematodinium (18SF2/Hem3R) | Haemolymph |
| MN057817 | Seq35 | D23_Feb | Hematodinium (18SF2/Hem3R) | Haemolymph |
| MN057818 | Seq36 | D31_Feb | Hematodinium (18SF2/Hem3R) | Haemolymph |
| MN057819 | Seq37 | P2_Mar | Hematodinium (18SF2/Hem3R) | Haemolymph |
| MN057820 | Seq38 | P7_Mar_F | Hematodinium (18SF2/Hem3R) | Haemolymph |
| MN057821 | Seq39 | P10_Mar | Hematodinium (18SF2/Hem3R) | Haemolymph |
| MN057822 | Seq40 | P17_Mar | Hematodinium (18SF2/Hem3R) | Haemolymph |
| MN057823 | Seq41 | P20_Mar | Hematodinium (18SF2/Hem3R) | Haemolymph |
| MN057824 | Seq42 | P22_Mar | Hematodinium (18SF2/Hem3R) | Haemolymph |
| MN057825 | Seq43 | P23_Mar | Hematodinium (18SF2/Hem3R) | Haemolymph |
| MN057826 | Seq44 | P25_Mar | Hematodinium (18SF2/Hem3R) | Haemolymph |
| MN057827 | Seq45 | P30_Mar | Hematodinium (18SF2/Hem3R) | Haemolymph |
| MN057828 | Seq46 | P35_Mar | Hematodinium (18SF2/Hem3R) | Haemolymph |
| MN057829 | Seq47 | P39_Mar | Hematodinium (18SF2/Hem3R) | Haemolymph |
| MN057830 | Seq48 | P41_Mar | Hematodinium (18SF2/Hem3R) | Haemolymph |
| MN057831 | Seq49 | D11_Mar | Hematodinium (18SF2/Hem3R) | Haemolymph |
| MN057832 | Seq50 | D12_Mar | Hematodinium (18SF2/Hem3R) | Haemolymph |
| MN057833 | Seq51 | D16_Mar | Hematodinium (18SF2/Hem3R) | Haemolymph |
| MN057834 | Seq52 | D20_Mar | Hematodinium (18SF2/Hem3R) | Haemolymph |
| MN057835 | Seq53 | D32_Mar | Hematodinium (18SF2/Hem3R) | Haemolymph |
| MN057836 | Seq54 | D35_Mar | Hematodinium (18SF2/Hem3R) | Haemolymph |
| MN057837 | Seq55 | P8_Apr | Hematodinium (18SF2/Hem3R) | Haemolymph |
| MN057838 | Seq56 | P24_Apr | Hematodinium (18SF2/Hem3R) | Haemolymph |
| MN057839 | Seq57 | P29_Apr | Hematodinium (18SF2/Hem3R) | Haemolymph |
| MN057840 | Seq58 | P35_Apr | Hematodinium (18SF2/Hem3R) | Haemolymph |
| MN057841 | Seq59 | P41_Apr | Hematodinium (18SF2/Hem3R) | Haemolymph |
| MN057842 | Seq60 | P45_Apr | Hematodinium (18SF2/Hem3R) | Haemolymph |
| MN057843 | Seq61 | P50_Apr | Hematodinium (18SF2/Hem3R) | Haemolymph |
| MN057844 | Seq62 | D6_Apr | Hematodinium (18SF2/Hem3R) | Haemolymph |
| MN057845 | Seq63 | D9_Apr | Hematodinium (18SF2/Hem3R) | Haemolymph |
| MN057846 | Seq64 | D10_Apr | Hematodinium (18SF2/Hem3R) | Haemolymph |
| MN057847 | Seq65 | D11_Apr | Hematodinium (18SF2/Hem3R) | Haemolymph |
| MN057848 | Seq66 | D15_Apr | Hematodinium (18SF2/Hem3R) | Haemolymph |
| MN057849 | Seq67 | D21_Apr | Hematodinium (18SF2/Hem3R) | Haemolymph |
| MN057850 | Seq68 | D46_Apr | Hematodinium (18SF2/Hem3R) | Haemolymph |
| MN057851 | Seq69 | D47_Apr | Hematodinium (18SF2/Hem3R) | Haemolymph |
| MN057852 | Seq70 | P22_May | Hematodinium (18SF2/Hem3R) | Haemolymph |
| MN057853 | Seq71 | P24_May | Hematodinium (18SF2/Hem3R) | Haemolymph |
| MN057854 | Seq72 | P27_May | Hematodinium (18SF2/Hem3R) | Haemolymph |
| MN057855 | Seq73 | P30_May | Hematodinium (18SF2/Hem3R) | Haemolymph |
| MN057856 | Seq74 | P32_May | Hematodinium (18SF2/Hem3R) | Haemolymph |
| MN057857 | Seq75 | P37_May | Hematodinium (18SF2/Hem3R) | Haemolymph |
| MN057858 | Seq76 | D4_May | Hematodinium (18SF2/Hem3R) | Haemolymph |
| MN057859 | Seq77 | D7_May | Hematodinium (18SF2/ITS R1) | Haemolymph |
| MN057860 | Seq78 | D8_May | Hematodinium (18SF2/Hem3R) | Haemolymph |
| MN057861 | Seq79 | D9_May | Hematodinium (18SF2/Hem3R) | Haemolymph |
| MN057862 | Seq80 | D42_May | Hematodinium (18SF2/Hem3R) | Haemolymph |
| MN057863 | Seq81 | D48_May | Hematodinium (18SF2/Hem3R) | Haemolymph |
| MN057864 | Seq82 | P16_Jun | Hematodinium (18SF2/Hem3R) | Haemolymph |
| MN057865 | Seq83 | P22_Jun | Hematodinium (18SF2/Hem3R) | Haemolymph |
| MN057866 | Seq84 | P38_Jun | Hematodinium (18SF2/Hem3R) | Haemolymph |
| MN057867 | Seq85 | P49_Jun | Hematodinium (18SF2/Hem3R) | Haemolymph |
| MN057868 | Seq86 | P50_Jun | Hematodinium (18SF2/Hem3R) | Haemolymph |
| MN057869 | Seq87 | D2_Jun | Hematodinium (18SF2/Hem3R) | Haemolymph |
| MN057870 | Seq88 | D12_Jun | Hematodinium (18SF2/Hem3R) | Haemolymph |
| MN057871 | Seq89 | D16_Jun | Hematodinium (18SF2/Hem3R) | Haemolymph |
| MN057872 | Seq90 | D18_Jun | Hematodinium (18SF2/Hem3R) | Haemolymph |
| MN057873 | Seq91 | D24_Jun | Hematodinium (18SF2/Hem3R) | Haemolymph |
| MN057874 | Seq92 | D28_Jun | Hematodinium (18SF2/Hem3R) | Haemolymph |
| MN057875 | Seq93 | D30_Jun | Hematodinium (18SF2/Hem3R) | Haemolymph |
| MN057876 | Seq94 | D31_Jun | Hematodinium (18SF2/Hem3R) | Haemolymph |
| MN057877 | Seq95 | D33_Jun | Hematodinium (18SF2/Hem3R) | Haemolymph |
| MN057878 | Seq96 | D35_Jun | Hematodinium (18SF2/Hem3R) | Haemolymph |
| MN057879 | Seq97 | D37_Jun | Hematodinium (18SF2/Hem3R) | Haemolymph |
| MN057880 | Seq98 | D46_Jun | Hematodinium (18SF2/Hem3R) | Haemolymph |
| MN057881 | Seq99 | P21_Jul | Hematodinium (18SF2/ITS R1) | Haemolymph |
| MN057882 | Seq100 | P31_Jul | Hematodinium (18SF2/Hem3R) | Haemolymph |
| MN057883 | Seq101 | P41_Jul | Hematodinium (18SF2/ITS R1) | Haemolymph |
| MN057884 | Seq102 | P42_Jul | Hematodinium (18SF2/Hem3R) | Haemolymph |
| MN057885 | Seq103 | D15_Jul | Hematodinium (18SF2/Hem3R) | Haemolymph |
| MN057886 | Seq104 | D16_Jul | Hematodinium (18SF2/Hem3R) | Haemolymph |
| MN057887 | Seq105 | D23_Jul | Hematodinium (18SF2/Hem3R) | Haemolymph |
| MN057888 | Seq106 | D34_Jul | Hematodinium (18SF2/Hem3R) | Haemolymph |
| MN057889 | Seq107 | D38_Jul | Hematodinium (18SF2/Hem3R) | Haemolymph |
| MN057890 | Seq108 | D42_Jul | Hematodinium (18SF2/ITS R1) | Haemolymph |
| MN057891 | Seq109 | P4_Aug | Hematodinium (18SF2/Hem3R) | Haemolymph |
| MN057892 | Seq110 | P14_Aug | Hematodinium (18SF2/Hem3R) | Haemolymph |
| MN057893 | Seq111 | P15_Aug | Hematodinium (18SF2/Hem3R) | Haemolymph |
| MN057894 | Seq112 | P21_Aug | Hematodinium (18SF2/Hem3R) | Haemolymph |
| MN057895 | Seq113 | P24_Aug | Hematodinium (18SF2/Hem3R) | Haemolymph |
| MN057896 | Seq114 | P31_Aug | Hematodinium (18SF2/Hem3R) | Haemolymph |
| MN057897 | Seq115 | P43_Aug | Hematodinium (18SF2/Hem3R) | Haemolymph |
| MN057898 | Seq116 | P45_Aug | Hematodinium (18SF2/Hem3R) | Haemolymph |
| MN057899 | Seq117 | P48_Aug | Hematodinium (18SF2/Hem3R) | Haemolymph |
| MN057900 | Seq118 | D1_Aug | Hematodinium (18SF2/Hem3R) | Haemolymph |
| MN057901 | Seq119 | D24_Aug | Hematodinium (18SF2/Hem3R) | Haemolymph |
| MN057902 | Seq120 | D25_Aug | Hematodinium (18SF2/Hem3R) | Haemolymph |
| MN057903 | Seq121 | D50_Aug | Hematodinium (18SF2/Hem3R) | Haemolymph |
| MN057904 | Seq122 | P6_Sept | Hematodinium (18SF2/Hem3R) | Haemolymph |
| MN057905 | Seq123 | P15_Sept | Hematodinium (18SF2/Hem3R) | Haemolymph |
| MN057906 | Seq124 | P19_Sept | Hematodinium (18SF2/Hem3R) | Haemolymph |
| MN057907 | Seq125 | D12_Sept | Hematodinium (18SF2/Hem3R) | Haemolymph |
| MN057908 | Seq126 | D44_Sept | Hematodinium (18SF2/Hem3R) | Haemolymph |
| MN057909 | Seq127 | P3_Oct | Hematodinium (18SF2/Hem3R) | Haemolymph |
| MN057910 | Seq128 | P18_Oct | Hematodinium (18SF2/Hem3R) | Haemolymph |
| MN057911 | Seq129 | P24_Oct | Hematodinium (18SF2/Hem3R) | Haemolymph |
| MN057912 | Seq130 | P30_Oct | Hematodinium (18SF2/Hem3R) | Haemolymph |
| MN057913 | Seq131 | D20_Oct | Hematodinium (18SF2/Hem3R) | Haemolymph |
| MN057914 | Seq132 | D33_Oct | Hematodinium (18SF2/Hem3R) | Haemolymph |
| MN057915 | Seq133 | D35_Oct | Hematodinium (18SF2/Hem3R) | Haemolymph |
| MN057916 | Seq134 | D45_Oct | Hematodinium (18SF2/Hem3R) | Haemolymph |
| MN057917 | Seq135 | D47_Oct | Hematodinium (18SF2/Hem3R) | Haemolymph |
| MN057918 | Seq136 | D49_Oct | Hematodinium (18SF2/Hem3R) | Haemolymph |
| MN049783 | Seq138 | Nov_1 | Hematodinium (HematF/HematR) | Seawater filter (eDNA) |
| MN049784 | Seq139 | Nov_2 | Hematodinium (HematF/HematR) | Seawater filter (eDNA) |
| MN049785 | Seq140 | Nov_3 | Hematodinium (HematF/HematR) | Seawater filter (eDNA) |
| MN049786 | Seq141 | Dec_1 | Hematodinium (HematF/HematR) | Seawater filter (eDNA) |
| MN049787 | Seq142 | Dec_2 | Hematodinium (HematF/HematR) | Seawater filter (eDNA) |
| MN049788 | Seq143 | Dec_3 | Hematodinium (HematF/HematR) | Seawater filter (eDNA) |
| MN049789 | Seq144 | Aug_3 | Hematodinium (HematF/HematR) | Seawater filter (eDNA) |
